# Structure-Based Reverse Vaccinology Failed in the Case of HIV Because it Disregarded Accepted Immunological Theory

**DOI:** 10.3390/ijms17091591

**Published:** 2016-09-21

**Authors:** Marc H. V. Van Regenmortel

**Affiliations:** UMR 7242 Biotechnologie et Signalisation Cellulaire, Université de Strasbourg-CNRS, 300, Boulevard Sébastien Brant, CS 10413, 67412 Illkirch Cedex, France; vanregen@unistra.fr; Tel.: +27-79-33-76-766

**Keywords:** antibody polyspecificity, continuous epitope, degeneracy of the immune system, discontinuous epitope, mimotope, plasticity of viral proteins, structure-based reverse vaccinology, rational vaccine design, paradigms in vaccinology, V3 loop of HIV-1 spikes

## Abstract

Two types of reverse vaccinology (RV) should be distinguished: genome-based RV for bacterial vaccines and structure-based RV for viral vaccines. Structure-based RV consists in trying to generate a vaccine by first determining the crystallographic structure of a complex between a viral epitope and a neutralizing monoclonal antibody (nMab) and then reconstructing the epitope by reverse molecular engineering outside the context of the native viral protein. It is based on the unwarranted assumption that the epitope designed to fit the nMab will have acquired the immunogenic capacity to elicit a polyclonal antibody response with the same protective capacity as the nMab. After more than a decade of intensive research using this type of RV, this approach has failed to deliver an effective, preventive HIV-1 vaccine. The structure and dynamics of different types of HIV-1 epitopes and of paratopes are described. The rational design of an anti-HIV-1 vaccine is shown to be a misnomer since investigators who claim that they design a vaccine are actually only improving the antigenic binding capacity of one epitope with respect to only one paratope and not the immunogenic capacity of an epitope to elicit neutralizing antibodies. Because of the degeneracy of the immune system and the polyspecificity of antibodies, each epitope studied by the structure-based RV procedure is only one of the many epitopes that the particular nMab is able to recognize and there is no reason to assume that this nMab must have been elicited by this one epitope of known structure. Recent evidence is presented that the trimeric Env spikes of the virus possess such an enormous plasticity and intrinsic structural flexibility that it is it extremely difficult to determine which Env regions are the best candidate vaccine immunogens most likely to elicit protective antibodies.

## 1. Introduction

The development of bacterial vaccines using the reverse vaccinology (RV) approach was pioneered by Rino Rappuoli about 15 years ago [1]. RV refers to the strategy of using bioinformatics analyses of entire bacterial genomes in order to identify all the surface-exposed proteins that the pathogen is able to express. These antigens are then produced by high-throughput technologies and tested for their immunoreactivity with patient sera and for their ability to induce protective antibodies. The strategy was called RV because the investigators operate in a so-called reverse manner, i.e., starting from the genome rather than from the organism, to discover which proteins are potential vaccine immunogens. Compared to the classical approach of fractionating bacterial extracts to identify a small number of potential vaccine antigens, RV makes it possible to evaluate hundreds of expressed bacterial proteins for their capacity to induce a protective immune response. Furthermore, RV allows immunogens to be tested even if the bacteria cannot be cultivated. In an effort to develop a Meningococcus B (Men B) vaccine, 350 open reading frames (ORFs) out the 2158 ones present in the Men B genome were cloned in *Escherichia coli* and the proteins were purified in sufficient amounts to immunize mice. A total of 90 previously unknown antigens were discovered in this manner, of which 28 were able to induce antibodies (Abs) that killed the bacteria [2] (pp. 225–241). In earlier studies using fractionated bacterial extracts, only 12 Men B surface antigens had been identified, of which only four induced Abs with bactericidal activity. By sequencing the entire genome of Men B, the complete antigenic repertoire of the organism could be analyzed, demonstrating the enormous potential of RV.

## 2. Genome-Based and Structure-Based Reverse Vaccinology

RV has been very successful for developing new bacterial vaccines [3] but has failed in the case of HIV vaccines. The main reason for this is that HIV particles contain only a small number of proteins useful for vaccination purposes, which require a particular tertiary or quaternary conformation to be effective vaccine immunogens. As a result, the RV strategies used in bacteriology and in virology are completely different. In virology, RV refers to the strategy of trying to generate a vaccine by determining the crystallographic structure of a complex between a viral epitope and a neutralizing monoclonal antibody (nMab). Instead of generating neutralizing Abs by immunization with a viral antigen, investigators use a so-called reverse approach by starting from the known structure of nMabs [4,5]. The nMab is then used as a template to reconstruct, outside the context of the native viral protein, the epitope recognized by the Ab using reverse engineering. The assumption is made that the reconstructed epitope designed to fit the nMab will have acquired the immunogenic capacity to induce a polyclonal Ab response endowed with the same neutralizing capacity as the nMab. Since the RV strategies used in bacteriology and virology are completely different, it has been suggested that they should be differentiated and called genome-based and structure-based RV, respectively [6].

This structure-based RV approach has been used in hundreds of attempts to develop an HIV-1 vaccine using as templates broadly neutralizing (bn)Mabs that recognized the major antigenic sites of HIV-1 [7,8,9]. Additional bnMabs have also been obtained from HIV-1 infected individuals by cloning antigen-specific memory B cells [10] and these Mabs made it possible to dissect numerous epitopes located in the HIV-1 Env protein such as the conserved CD4-binding site, the CD-4 induced antigenic site and the V3 antigenic site, as well as epitopes located in the membrane proximate external region (MPER) of the HIV-1 gp41 protein. Numerous strategies were developed for improving the antigenic reactivity of these epitopes [11,12] and although some of the engineered epitopes reacted better with the bnMabs used as templates, none of them were effective immunogens able to induce broadly neutralizing antibodies.

## 3. Structural Vaccinology

The large number of protein antigens that can be studied by genome-based RV makes it possible to select native bacterial proteins that will be effective vaccine immunogens. In some cases, knowledge of the 3D structure of certain bacterial proteins helps to improve the efficacy of bacterial vaccines, as demonstrated with the factor H-binding protein of Men B [13] and the pili of group B Streptococcus [14]. This led to the use of the term “structural vaccinology” in the field of bacterial vaccines [15].

In virology, structural vaccinology had been introduced about 10 years earlier [4] and was called RV, although structure-based RV is probably a better term for it. Following the dramatic world-wide expansion of the AIDS epidemic, considerable funding became available for studying HIV-1, and large teams of investigators embarked on the search for a much needed HIV-1 vaccine.

In view of the considerable knowledge that was available regarding the structure of immunoglobulins and of HIV-1 [16,17,18,19,20,21], many scientists believed that structure-based RV was a realistic strategy for developing an HIV-1 vaccine. However, after more than a decade of intensive research funded by billions of dollars, the outcome is very disappointing since little tangible progress has been made in the search for a preventive HIV-1 vaccine [11,12,22,23]. 

There are many reasons why the successful strategies used for developing viral vaccines in the past failed in the case of HIV-1 [24,25,26,27]. The natural immune response in HIV-1 infected individuals, for instance, does not clear the infection, which means that a successful vaccine must achieve something that the human immune system (IS) is not capable of doing when it encounters the virus. There are other major impediments namely that (1) HIV-1 integrates into the host genome and conceals the virus from immune recognition; (2) the virus exhibits an enormous antigenic variability and progressively destroys the IS; and (3) in order to acquire sufficient neutralizing capacity, HIV-1 Abs need a much longer affinity maturation process than do Abs elicited by other viruses, with the result that protective Abs usually appear only after about two years [28]. It is not possible during such a long Ab maturation process to use the structure-based RV approach since it is not clear which of the many intermediate Abs in the maturation pathway should be selected as templates for engineering candidate vaccine epitopes. Reverse vaccinologists base the design of vaccine immunogens solely on the observed structural complementarity between one epitope and one bnMab and they disregard the fact that numerous complex biological mechanisms and components of the IS are actually involved in the production of antibodies. Once antigens or individual epitopes are introduced in an IS, they become known as immunogens because they trigger the IS to start synthesizing antibodies that may or may not recognize the immunogen [29]. When it was established that HIV-1 epitopes recognized by affinity-matured bnAbs derived from HIV-1-infected individuals did not bind the germline predecessors of these antibodies [30,31], it became clear that a lengthy process of antibody affinity maturation would have to be taken into account for obtaining neutralizing anti-HIV-1 antibodies. This meant that the design of HIV vaccines using RV was doomed and a huge, novel research effort was started in an attempt to unravel individual Ab affinity maturation pathways that lead from non-neutralizing to neutralizing Abs [32,33,34]. The general aim was to drive human immune responses towards highly mutated anti-HIV bnAbs, using sequential immunizations with various Env immunogens [35]. In view of the stochastic nature of mutations and the huge number of individual maturation pathways that exist [31], it is not at all clear whether this would make it possible to identify a small number of effective immunogens that could be employed for large scale human vaccination campaigns. Since this new vaccination strategy [35] has nothing in common with the RV approach that aims to transform HIV-1 epitopes of known structure directly into vaccine immunogens, it will not be further discussed in this review, which only analyzes why the RV approach used for several years failed to deliver an effective HIV-1 vaccine.

## 4. Structure and Dynamics of Epitopes and Paratopes

The B cell epitopes of proteins are the regions that are recognized by the binding sites (i.e., the so-called paratopes) of Ab molecules present either in their free form in serum or as membrane-bound B cell receptors (BCRs). B cell epitopes must be distinguished from T cell epitopes, which are proteolytically cleaved peptides of the antigen that interact with T cell receptors. In the present review, B cell epitopes will simply be referred to as epitopes. 

Protein epitopes are usually classified as continuous or discontinuous, depending on whether the amino acids present in the epitope are contiguous in the peptide chain or not. X-ray crystallographic analysis of epitopes bound to paratopes revealed that the vast majority of protein epitopes are discontinuous and consist of residues located on two to five separate chain segments that are brought together on the protein surface by the folding of the peptide chain. This distinction between the two types of epitopes may lead one to believe that amino acid residues are the entities involved in Ab-antigen recognition, although it is at the level of individual atoms that interactions take place [36].

### 4.1. Discontinuous Epitopes

**Figure 1:**
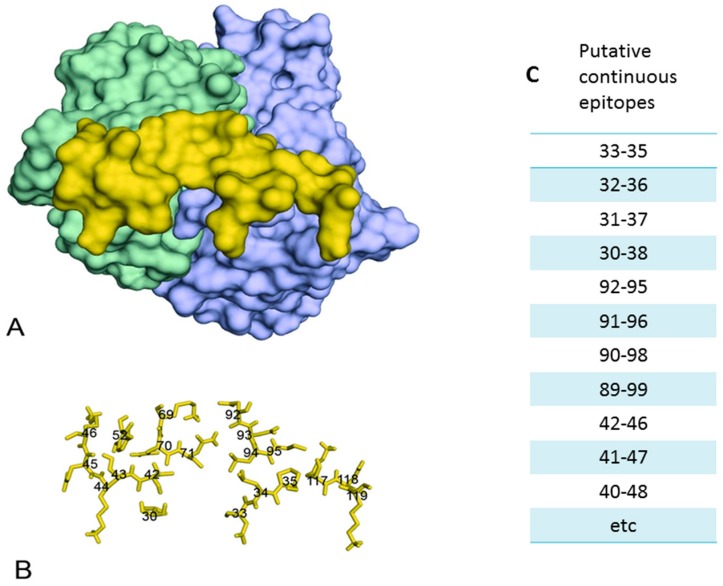
Discontinuous epitope of the outer surface protein A of the spirochete *Borrelia burdorferi* elucidated by X-ray crystallography from a complex with Mab 184.1. (**A**) Outline of the epitope in yellow; (**B**) position in space of the residues comprising the epitope. This set of residues cannot be isolated as such from the protein to demonstrate that it possesses binding activity in its own; (**C**) Parts of the discontinuous epitope and other peptide segments of the protein that may be able to bind Mab 184.1, in which case they would be called continuous epitopes (courtesy of Pernille Haste-Andersen, Danish Technical University).

Figure 1 shows the structure of a discontinuous epitope present in the outer surface protein A (OspA) of the spirochete *Borrelia burdorferi*, the etiological agent of Lyme disease. The structure of this epitope was elucidated by X-ray crystallography of OspA complexed with the Fab fragment of the mouse Mab 184 [37]. The epitope consists of residues 30, 33–35, 42–46, 52, 67–71, 92–95, and 117–119, which are in contact with residues of the paratope. Atomic groups in the residues that form the seven sections of the epitope are not held together by internal chemical bonds and they are recognized by antibodies only because the entire peptide chain acts as a scaffold. A discontinuous epitope lies in a molecule and acts like a molecule although it is not actually a molecule [38] (p. 273). If the tertiary structure is perturbed—for instance, when the protein is denatured—the epitope ceases to exist. Discontinuous epitopes in a native protein are therefore only defined structurally by the set of atoms that make contact with residues of the Ab. A contact is said to occur if the interatomic distance between atoms in the epitope and paratope is 3.5 to 4.5 Å, depending on the chemical nature of the interaction [39].

When the epitope is represented as a disembodied set of residues (Figure 1B) it is clear that this set of residues cannot be isolated or extracted from the OspA protein as a functional unit possessing binding activity of its own. Discontinuous epitopes are thus defined only structurally by atomic contacts with a paratope and not by showing experimentally that a series of non-contiguous residues possess binding activity outside the context of the native protein. For this reason, the functional capacity of a discontinuous epitope of HIV-1 to induce Abs can only be assessed by using as immunogen the native protein in which the epitope is embedded. Since such a procedure always gives rise to a heterogeneous immune response against the many epitopes present in the protein, it is impossible to study separately the immunogenic activity of one discontinuous epitope.

X-ray crystallographic analysis of Ab-antigen complexes provides only static pictures of epitopes and paratopes. A molecular structure is always the result of selective attention to the visual experience of an object at a specific time [40] and thinking in terms of static instead of dynamic structures tends to conceal the role that mutual adaptation in the Ab and antigen plays in facilitating their interaction [41]. Proteins and immunoglobulins are fairly dynamic molecules [42] and the plasticity of epitopes and paratopes has led to them being described as flexible keys interacting with adjustable locks [43].

It is well established that the structures visualized in epitope–paratope complexes are often very different from the structures of the binding sites in the free molecules before they were altered by the mutual adaptation and induced fit that occur when the two partners interact [44,45]. The particular epitope structure observed in an epitope–paratope complex therefore does not necessarily correspond to the immunogenic structure recognized by BCRs during the immunization process that gave rise to the Ab; such an epitope identified in a complex may thus not be the best structure that should be mimicked in a vaccine. 

**Figure 2:**
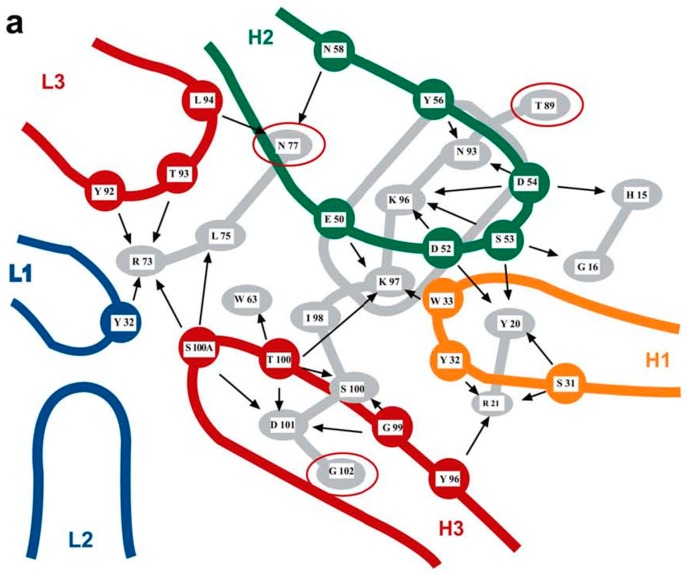

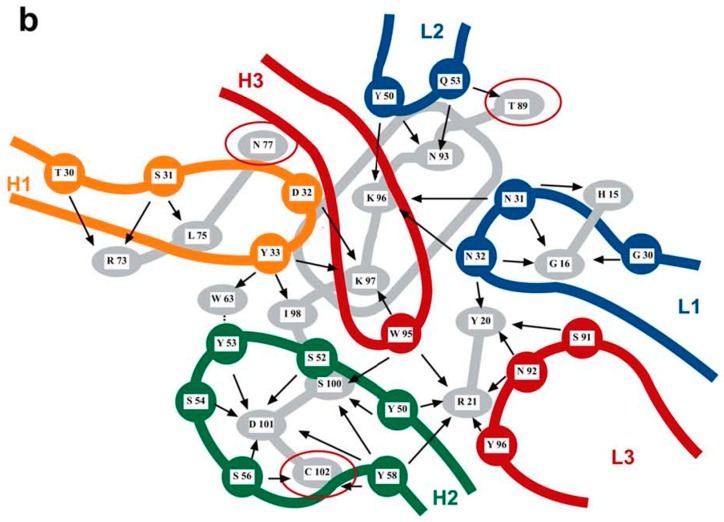
The same residues of an antigen can contribute to different overlapping discontinuous epitopes. Two overlapping discontinuous epitopes of lysozyme are recognized by Mabs F9-13.7 (**a**); and HyHEL10 (**b**) elucidated by X-ray crystallography. Thirteen residues of lysozyme (in gray) are recognized by both antibodies, albeit with different bonding patterns. The rounded rectangle in gray represents the lysozyme α-helix. The two sets of CDRs are shown in color and have different orientations on the lysozyme surface. Three residues (N77, T89, and G102 highlighted with red circles) are not shared by the two epitopes. Intermolecular contacts are shown by arrows. Mab HyHEL10 forms a salt bridge between lysozyme K97 and residue D32 of the H1 antibody loop. Mab F9-13.7 forms salt bridges between lysozyme residues K97, K96, and H15 and respectively residues E50, D52, and D54 of the H2 antibody loop (adapted from Lescar et al., 1995 [47]).

Since the entire accessible surface of a protein is a continuum of potential epitopes, the same residues may contribute to several overlapping discontinuous epitopes recognized by different Abs. This is illustrated in Figure 2 for two Mabs that recognize a very similar epitope in lysozyme by using two completely different paratopes presenting no similarity in their chemical bonding patterns with the epitope. Since the epitope nature of a set of amino acids can only be established when an Ab able to bind to it has been found, an epitope is not an intrinsic structural feature of a protein that could be identified in the absence of a particular interaction with a paratope. Similarly, since a paratope requires the existence of a complementary epitope in order to acquire a recognizable identity, both epitopes and paratopes are relational entities defined by their mutual complementarity [46]. This means that the two Mabs in Figure 2 do not recognize the same epitope.

The number of epitopes in a protein can be estimated from the number of different Mabs that can be raised against it. This number was found to be 115 epitopes for insulin [48] and more than a thousand for the BLyS molecule [49]. This relational nexus means that analyzing the antigenicity of a protein amounts to analyzing the size of the immunological repertoire of the host immunized with the protein [46].

The identical viral subunits that are assembled in virions and in spikes embedded in viral membranes [50] give rise to a quaternary protein structure that creates discontinuous epitopes by the juxtaposition of residues from neighboring subunits that Abs recognize as a single epitope. Such epitopes, which have been called neotopes [51,52], have been shown to be present in the capsids and membrane proteins of many viruses including HIV-1 [53,54,55]. Neotopes can also arise from conformational changes induced in the subunits by intersubunit interactions. Since the quaternary structure of viral proteins is sensitive to small changes in the chemical environment [56], neotopes in HIV can be transient epitopes capable of assuming different structures alternating between open and closed conformations [57]. It is likely that such conformational variability will facilitate induced fit adjustments and BCR recognition although there is also evidence that immunogenicity can be enhanced when epitope flexibility is either increased or decreased [58,59,60].

Since many crystal structures of epitope–paratope complexes have been determined to a resolution of 30 Å or less, the precise nature of the atomic interactions is usually not established. This may partly explain why protein crystallographers for a long time believed that water was actually extruded from epitope–paratope interfaces [61]. Water of hydration surrounds all protein molecules [36] but these water molecules are not necessarily expelled from the contact interface because of the imperfect complementarity between epitopes and paratopes. As a result, water molecules may contribute H-bonds to the interaction between the two partners [62,63], although their contribution to the binding energetics is difficult to quantify [64].

**Figure 3:**
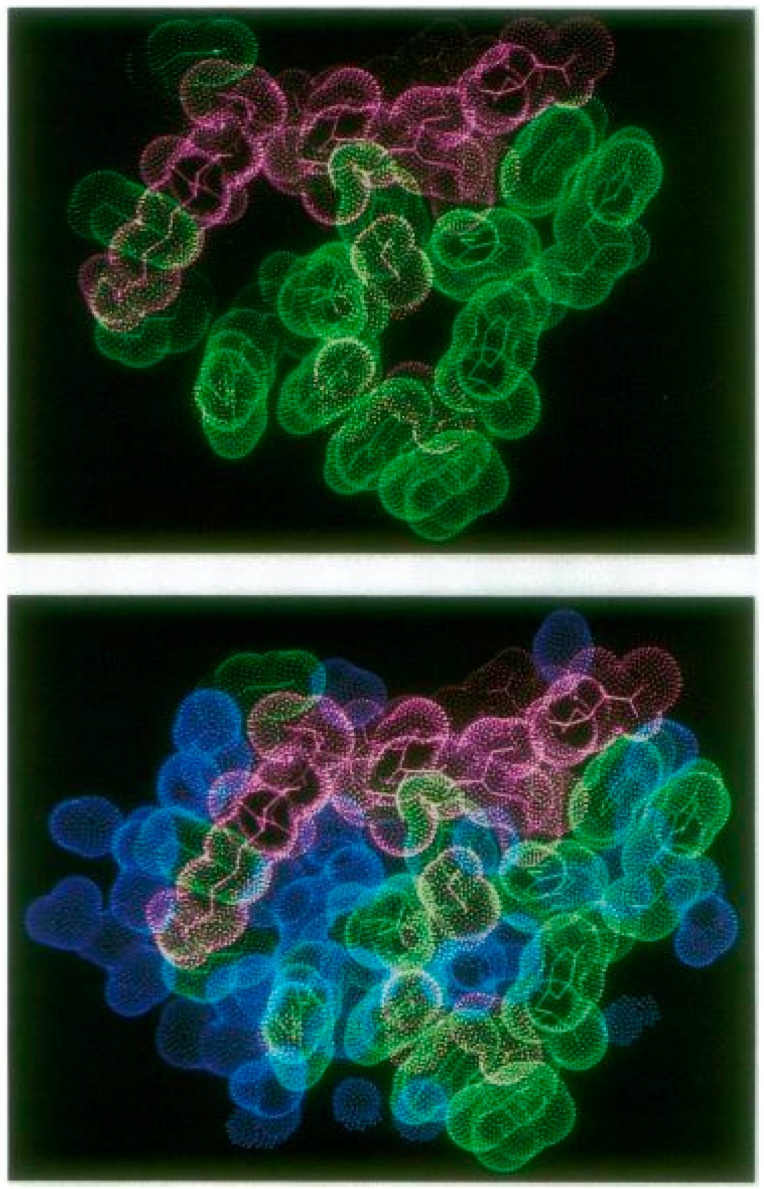
3D structure at 1.8 Å resolution of the anti-lysozyme Fv fragment of Mab D1.3. **Upper** frame: van der Waals surface of antibody residues (in **green**) interacting with residues of lysozyme (in **pink**); **Lower** frame: the same model showing cavities and channels filled with water molecules (in **blue**) after complex formation. There are more water molecules at the interface than in the free binding sites and they contribute H-bonds that stabilize the complex. The association is not driven by an hydrophobic entropy effect arising from the extrusion of water from the interface but by a large negative enthalpy arising from H-bonds and van der Waals interactions (Bhat et al., 1994 [65]).

In a seminal study of the complex between lysozyme and the Fab fragment of Mab D 1.3 in its antigen-bound form, compared to its free form, at an improved resolution of 1.8 Å, Roberto Poljak and his collaborators established that 48 water molecules were present at the epitope–paratope interface, which is larger than the number of water molecules associated with the unbound sites [65]. Both buried and exposed water molecules establish links between the antigen and Ab and contribute to the chemical complementarity between the two interacting surfaces (Figure 3), leading to decreased mobility in the Ab structure upon complex formation.

The history of how these results became rapidly accepted is interesting. The journals *Nature* and *Science*, as custodians of scientific orthodoxy, had refused to publish the paper because it contradicted the theory, universally accepted by crystallographers at the time, that water molecules are always extruded from antigen–antibody interfaces, leading to increased entropy. However, for more than a year before the results were finally published, Poljak had presented his data at several international meetings and the quality of his experimental data (Figure 3) together with his new interpretation of Ab-antigen interactions convinced many crystallographers. Very soon after the 1994 publication [65], such results became acceptable and were published by other groups [66,67].

### 4.2. Continuous Epitopes

Any short linear peptide fragment of a protein able to bind to anti-protein antibodies is called a continuous epitope of that protein. This terminology is unfortunate because it may give the impression that these epitopes are present as such in the native protein although short peptide fragments very rarely retain the conformation present in the corresponding part of the folded protein [46]. Since short peptides of 3–5 residues corresponding only to portions of a discontinuous epitope are often able to bind weakly to antibodies raised against the discontinuous epitope, such peptides will often be called continuous epitopes [68]. For instance, peptide fragments of OspA corresponding to residues 42–46, 40–48, or 40–50 of the molecule (Figure 1B) could easily be identified as continuous epitopes because they all contain five contiguous residues of the OspA sequence, which is sufficient to give rise to a weak cross-reaction with the OspA antibody [69] (pp. 1–27) [70]. It has been suggested that the majority of continuous epitopes described in the literature may correspond to unfolded regions of denatured proteins and are therefore not genuine epitopes of native proteins [71]. It can, indeed, never be excluded that the protein antisera used for measuring the antigenic reactivity of peptides contain Abs to denatured molecules that were present in the protein preparation used for eliciting the antisera. In the reciprocal assay when Abs raised against peptides are tested for their ability to react with the cognate protein, it may appear that the Abs also recognize the protein molecules because these have become denatured when they are adsorbed to plastic in a solid phase immunoassay. The initial claim that individual immunizations with 20 residue-long synthetic peptides corresponding to the entire sequence of influenza hemagglutinin were all able to induce antibodies that cross-reacted with the viral protein stimulated considerable interest in the possibility of using synthetic peptides as vaccines [72]. However, it was subsequently shown that such misleading results were obtained because the viral antigens used in the solid-phase immunoassay had become denatured by adsorption to plastic [73].

More than a thousand linear peptides corresponding to continuous epitopes have been studied over many years as potential synthetic peptide vaccines against numerous pathogens and it is remarkable that by 2006 not a single peptide had passed phased III clinical trials and was marketed as a human vaccine [74]. It is therefore astonishing that many investigators still try to develop HIV-1 vaccines starting from continuous epitopes, especially because it is well-established that most nAbs against HIV-1 recognize discontinuous epitopes.

In order to ascertain whether antibodies elicited by peptides corresponding to small regions of a protein truly recognize the native form of the protein, it is necessary to test these antibodies in a liquid-phase immunoassay that preserves the native conformation of the protein [75]. Thousands of continuous epitopes listed in epitope databases have been identified only on the basis of their ability to cross-react with protein antibodies in a variety of immunoassays and not by showing that each residue in the peptide made contact with residues of a paratope [76,77]. Since the criterion used for identifying a continuous epitope is only its binding activity, the exact structure of the epitope is never clearly defined [78] (pp. 1–78). When epitopes are defined structurally by crystallography they comprise about 15 residues whereas if they are defined functionally by binding assays, they appear to be much smaller [79]. When peptides of decreasing size are tested for binding activity, the smallest peptide that retains a significant level of reactivity is sometimes given the status of epitope although it may be as short as a pentapeptide or a tripeptide [69,80,81] (pp. 265–310). This agrees with the finding that when small haptens of increasing size are tested for their ability to bind to a range of Mabs, a maximum binding affinity of 10^10^ M^−1^ is observed when the hapten molecular weight reaches a value of 450, which corresponds to about four amino acid residues [82,83] (pp. 357–372). All these finding have led to the conclusion that most studies that analyze the antigenic structure of proteins by binding assays do not focus on antigenicity per se but on the phenomenon of cross-reactive antigenicity between proteins and short peptides [79].

### 4.3. Mimotopes

The term mimotope was coined by Mario Geysen [84] to refer to a peptide that binds a particular Ab but possesses little or no sequence similarity with the protein antigen used to raise the Ab. Mimotopes are usually identified by testing combinatorial peptide libraries and selecting peptides that bind anti-protein Abs. The phenomenon of hydropathic complementarity (see Section 4.4) explains why peptides with little or no sequence similarity may nevertheless be able to react with the same Ab.

To qualify as a mimotope, the peptide in addition to binding to an anti-protein Ab should also be capable of eliciting Abs that recognize the epitope being mimicked [46]. The reason why this is a requirement is that a peptide may bind to a paratope in a polyspecific Ab (Section 4.4) that is not the same as the paratope that initially selected the mimotope from a peptide library.

Many mimotopes of HIV-1 epitopes have been isolated from peptide libraries but none of them induced nAbs when they were used as immunogens [85,86,87]. Peptide analogs that retain the original hydropathic complementarity present in a continuous epitope but no longer possess any sequence similarity with it may still bind to the same Ab, which explains the binding activity of certain mimotopes (see Section 4.4).

### 4.4. Paratopes

Paratopes are the binding sites of Abs that recognize epitopes. IgG immunoglobulins possess two identical binding sites formed by six surface loops located at the tip of the two Fab fragments of the IgG [88] (pp. 23–36), and comprising 50–70 hypervariable residues distributed over six complementarity determining regions (CDRs) in the variable domains of the heavy and light immunoglobulin chains. Each of the two Ig binding sites contains several smaller overlapping or non-overlapping subsites of 10–20 residues, called paratopes, which are the regions that bind to different epitopes of an antigen. As a result, an antibody can never be monospecific for a single epitope because the surface of one paratope represents only about 20%–35% of the total surface of the entire Ig binding site [89]. Steric hindrance usually prevents two antigens from binding simultaneously to the same Ig binding cleft, but when two paratope subsites do not overlap, two small antigens or haptens may be able to bind simultaneously to the same antibody molecule [90]. The overall structure of a paratope built up from residues present in different hypervariable loops is reminiscent of the structure of a discontinuous epitope that always consists of residues located in separate surface regions of a protein molecule. Since small peptides corresponding to a single CDR loop of an antibody are sometimes able on their own to bind the cognate antigen, they can be viewed as continuous paratopes, analogous to continuous epitopes [91]. The ability of some continuous epitopes to bind continuous paratopes is due to the type of hydropathic complementarity that is usually observed between the sense and antisense peptides encoded by complementary sense and antisense messenger RNAs [29,92,93,94]. Hydropathic complementarity arises from an inverted hydropathic pattern in two short peptide sequences and is caused by the attraction that exists between hydrophilic and hydrophobic groups [29].

Recent work by the group of Ofran in Israel has considerably improved our understanding of the structural basis of antibody specificity [95,96,97]. By analyzing 140 antibody–antigen complexes, they demonstrated the contributions of individual Ig binding loops to the overall binding specificity of antibodies and clearly established that there is no noticeable difference between the amino acid residue composition of epitopes and the composition of the entire protein surface [29,97]. This finding explains why innumerable attempts to predict epitopes in proteins using amino acid propensities such as surface accessibility or hydrophilicity were notoriously unsuccessful [98] because these prediction methods which concentrated on continuous epitopes assumed that certain surface residues possessed a superior intrinsic capacity for binding any potential antibody [29,78]. Prediction methods for discontinuous epitopes are also very limited because these methods are unable to identify the complete set of residues from distant parts of the protein sequence that needs to be assembled in a precise configuration in order to possess the antigenic and immunogenic activity of the epitope [29,99,100,101]. Antibody polyspecifity is the ability of an antibody molecule to bind a large variety of epitopes present in different antigens, and is caused by the presence in each Ab binding site of different paratopes that possess considerable conformational plasticity and flexibility. Several mechanisms of conformational adaptation within the antibody combining site allow a paratope to bind to different epitopes [102], which explains why most Abs derived from Ab germline genes are highly polyreactive and are able to react with a variety of self-antigens such as DNA, cytoskeleton proteins, carbohydrates as well as bacterial and viral antigens [103,104,105,106]. During somatic hypermutation and the resulting Ab maturation, the flexibility of paratopes tends to decrease as their affinity increases [42,107]. During normal human B cell development, many such Abs are deleted from the Ig repertoire in the bone narrow before they enter the mature B cell compartment [108] although as many of 20% of the Abs subsequently produced by mature human B cells may still be polyreactive and self-reactive [109]. Such findings put into question the validity of the need for absolute self–nonself discrimination as the primary factor that best explains the evolutionary origin of the IS [110,111].

## 5. The So-Called Rational Design of HIV-1 Vaccine Immunogens

The term design refers to the deliberate conceiving and creation of a novel object or process by an intelligent being. It is widely believed today that rational design is the best strategy for developing new drugs and vaccines [112,113] and that it is applicable to HIV-1 vaccine development [5,114,115,116,117].

The term “rational” is applied to modern vaccine design in the way the term is used in “rational drug design” i.e., for describing how candidate molecules are designed to fit the 3D structure of a biological target or receptor so that they will bind with high affinity and selectivity to it and inhibit a biological function [118]. 

As stated by Amzel in 1998 [119]: “One of the goals of research in biotechnology is to transform the process of developing a drug from a trial-and-error empirical operation into a rational structure-based process”. This computer-assisted approach based on molecular docking [120] succeeds because the complementarity between drugs and their target molecules is fairly unique. This explains, for instance, the remarkable success in developing the antiretroviral drugs that inhibit various HIV-1 enzymes [121,122]. 

In contrast, molecular recognition processes between an antigen and the many Abs that are able to bind to it are much less specific than drug-receptor interactions, mainly because of the conformational flexibility of paratopes, the degeneracy of the immune system and the polyspecificity of antibodies [42,123]. This allows an individual IS to recognize virtually any antigenic motif it may encounter [124,125,126].

### 5.1. What Do Vaccinologists Actually Do When They Claim to Be Rationally Designing an HIV-1 Vaccine?

Doing something by design is doing it intentionally; believers in “intelligent design” accept, for instance, that an intelligent deity designed all living organisms and that this explains the presence of life on our planet. Scientists today no longer believe that living organisms and their biological functions were designed by the preconceived plan of a designer rather than being shaped by the filter and pressure of Darwinian natural selection [127]. It seems equally inappropriate to use design terminology for describing the activities of vaccinologists when they attempt to develop an HIV-1 vaccine by studying the structure of antibodies able to neutralize the virus. What is of course feasible is to improve the binding complementarity present in one epitope–bnMab pair by either designing an HIV-Env epitope that binds better [128] or a paratope that binds better [129]. However, in both cases this amounts only to designing improved binders and not immunogens. Although the antigen binding capacity (i.e., the antigenicity) of an Env epitope may sometimes be improved, this is not the case for the ability of that epitope to elicit protective Abs (i.e., its immunogenicity) when it is used for immunization. The claim that what is being rationally designed is actually an HIV-1 vaccine immunogen instead of an antigen [114,117] arises because antigenicity and immunogenicity are confounded and the unwarranted assumption is made that when an epitope reacts strongly with a paratope present in the bnMab, this epitope will also be able to elicit similar neutralizing antibodies in an immunized host. Antigen design is simply masquerading as immunogen design since there is no justification for the belief that antigenic reactivity necessarily entails an immunogenic capacity to produce neutralizing Abs similar to the Mab that was used as template for designing the antigen [130,131].

### 5.2. Rational Vaccine Design without a Designer Is Only a Metaphor

In order to rationally design an HIV-1 vaccine, a designer would need to know the immune correlates of protection; these, however, are identifiable only after an effective vaccine has been developed. In the case of Darwinian evolution, Ayala [132] tried to rescue design as an explanation by suggesting that it is possible to have “design without a designer” although this simply encourages the use of metaphors of goal-directed teleology for describing natural biological processes in terms of design and purposes [127,133].

It has also been claimed that HIV-1 needs to continuously develop new strategies and mechanisms in order to escape IS defences, and that this amounts to a momentous evolutionary battle between virus and host [134]. However, such metaphoric language implies that the virus is able to develop new goal-directed mechanisms for winning a battle, whereas it is only the occurrence of stochastic mutations in the reverse transcriptase enzyme of the virus that is responsible for the persistent chronic infection [135].

Vaccinologists should not claim that they rationally design an HIV-1 vaccine when they are only improving the antigenic binding capacity of viral proteins. The neutralizing efficacy of Abs obtained by vaccination is due to the presence in the host IS of numerous biological and regulatory mechanisms that are unrelated to the actual epitope–paratope recognition process, which is the only parameter that the vaccine designer takes into account and tries to control [130,136]. Since it is not possible by structure-based RV to alter the activity of an IS so that it will produce nAbs, rational vaccine design without a designer, is best relegated to the sphere of poetic, metaphorical language.

## 6. Paradigms in HIV Vaccinology

The term paradigm was introduced by the philosopher and historian of science, Thomas Kuhn, in his 1962 book *The Structure of Scientific Revolutions*, [137], to refer to the conceptual framework within which scientists think and operate at a given time. Paradigms in vaccine research are usually based on recognized past vaccine achievements, since these provide an indication of which experiments might be conducted and what responses could be expected.

Investigators operating within a shared paradigm are often unaware of the many presuppositions, assumptions and conjectures that underlie their research agenda. When they obtain results that differ from what they expected, they will tend to formulate new ad hoc hypotheses to resolve contradictions between their observations and what they had predicted would happen [138]. However, they rarely conclude that the paradigm has been refuted and must be abandoned. Kuhn argued that when scientists obtain unexpected results, they remain committed to an accepted paradigm because they view it as a coherent conceptual framework that has been successful in the past. Only if a large number of anomalous results keep accumulating over many years may they lose their confidence in a given paradigm and eventually replace it by another one, giving rise to what has been called a paradigm shift. Kuhn [137] described such a change by saying that a long period of normal science is then followed by a short period of revolutionary science. 

When vaccinologists do not obtain the results they expected, they do not abandon a paradigm because they do not consider that the main purpose of their experimental work is to confirm the validity of the presuppositions inherent in the paradigm they adhere to. They will thus not follow the injunction of Karl Popper [139] that the primary aim of an investigator must be to try to disprove or falsify hypotheses or theories rather than to prove them. It is well known that scientific observations and empirical experimentation are never able to logically prove the correctness of a theory although they can sometimes refute it. When vaccinologists believe that they will achieve a particular research objective by following their chosen paradigm, they will disregard the possibility that some of their assumptions may be incompatible with current immunological theory (see Section 7). In the field of HIV vaccine research there is evidence that certain paradigms, for instance the assumption that successful results obtained previously with other viruses can be replicated with HIV, have led investigators astray and made them pursue strategies that were unlikely to be successful.

Paradigms come in different forms and some can be applicable to many viruses, whereas others are more restricted in scope. The first paradigm that guided HIV vaccine research in the years 1985 to 2003 was based on the belief that immunization with HIV-1 Env recombinant proteins would succeed in eliciting neutralizing Abs that would confer protection against infection. When this was found not to be the case, a new paradigm based on cell-mediated immunity was followed for a few years but was also abandoned when vaccine trials showed no efficacy [140,141]. 

A third paradigm based on the expectation that structure-based RV would be able to identify HIV-1 Env epitopes capable of eliciting broadly neutralizing Ab responses was proposed in 2002 [4] and pursued for several years but did not succeed in producing effective vaccine immunogens [11,22,23,130,142].

The R144 vaccine trial started in Thailand in 2004 combined a poxvirus-vectored gp120 env prime with a gp120 protein boost and followed a mixed paradigm that was widely condemned as ill-conceived [143,144]. However, it turned out to be the first human vaccine trial that led to some limited reduction in HIV-1 acquisition [145,146] illustrating the vagaries of paradigm selection.

Another paradigm of a broader nature has become popular in recent years because many investigators believed that we possess too little knowledge of the human IS and of HIV-1 immunopathology to be able to develop an effective HIV vaccine. This led to the view that progress would occur only if large-scale basic research programs in immunology were undertaken [27,147]. This paradigm finds its origin in the belief prevalent since the middle of the last century [148] that all technological innovations are the result of applied research made possible by previous curiosity-driven basic research that is undertaken without any consideration of potential use. Such a belief is less prevalent today because a tight separation between basic and applied research is considered to be somewhat artificial and it is accepted that research can be driven simultaneously by a quest for fundamental understanding and by considerations of use for solving a practical problem. According to the so-called quadrant model of scientific research [149], a commitment to study various properties of the IS does not exclude a commitment to try to control HIV-1 infection by vaccination [150,151]. In order to understand how a technological innovation such as a new vaccine becomes possible, it is necessary to distinguish between two types of human knowledge [152]:

(1) Knowing what is the case, which leads to factual knowledge (also called propositional knowledge) that takes the form of true statements concerning an established scientific fact. New factual knowledge is always *a discovery* that reveals something that existed all along but was unknown to anybody.
(2) Knowing how to do something of practical utility that leads to new prescriptive knowledge in the form of an *invention* that makes it possible to do something that was previously not feasible.

Factual knowledge is always derived from basic research whereas prescriptive knowledge, which could take the form of an innovation such as a new vaccine, always requires applied research to demonstrate how nature and natural phenomena can be successfully manipulated and controlled. This is illustrated, for instance, by the fact that factual knowledge concerning the contribution of long CDR-H3 regions of Abs to the exceptional neutralizing ability of certain anti-HIV-1 bnMabs [153,154] was of little help for developing a vaccine because there was no prescriptive knowledge available on how such Abs could be elicited by immunization [131].

In his book *Representing and Intervening*, Ian Hacking [155] pointed out that scientists obtain prescriptive knowledge only when they successfully intervene empirically in the system under investigation because this is the only way for them to demonstrate that they are indeed able to control it. The complexity of the IS makes it impossible to fully “understand” it in a reductionist manner since only a few causal links can be revealed between a small number of the numerous individual constituents of the system [156,157,158]. Biological science does not exhibit the law-like regularities found in the physical sciences and biologists are only able to partly explain phenomena by positing mechanisms and making predictions about their occurrence. However, they do not fully understand the innumerable interconnections that exist between all the parts of a complex system [130,159]. 

On the other hand, if vaccinologists succeed in intervening in an IS and achieve protective immunity against virus infection, this newly acquired prescriptive knowledge will allow them to make reliable predictions about the likely outcome of a vaccination trial. In other words, the empirical development of an effective vaccine must take place when the mode of action of an ideal HIV-1 vaccine has not yet been elucidated using factual knowledge obtained by previous basic research in immunology. Increasing our factual knowledge of immunological phenomena in no way eliminates the need to develop empirically the prescriptive knowledge required for vaccine development [22,142].

## 7. An Effective HIV-1 Vaccine Cannot Be Developed by Disregarding Accepted Immunological Theory

### 7.1. Facts, Hypotheses, Laws, Theories, and Empirical Experimentation

Alan Chalmers, in his popular book translated into 15 languages entitled *What is This Thing Called Science?* [160] suggested that “science is derived from the facts”, the facts being claims about the world that can be directly established by a careful, unprejudiced use of the senses. The term “fact” is somewhat ambiguous because it can refer to a statement that expresses a certain observable state of affairs or to the state of affairs itself. Scientific facts correspond to objective and verifiable observations that are often collected in order to build or reject hypotheses that could explain the occurrence of a certain phenomenon. A hypothesis may be nothing more than guesswork, a hunch or a conjecture, which is usually the case for the assumptions that underlie a paradigm. Scientific experiments are never able to “prove” that a hypothesis is correct although they can sometimes refute a mistaken hypothesis. Experiments may also fail to reject it, which means that the hypothesis could be correct but without any certainty [161,162] (pp. 32–41). Absolute proof in the mathematical sense or in the logical sense of deduction can never be derived from empirical, experimental science.

It is commonly believed that we derive facts simply by observing the world but this would mean that our visual perceptions require no interpretation and are not influenced by prior-knowledge or by a particular conceptual framework. That this is not the case is shown, for instance, by the belief, prevalent until the seventeenth century, that the earth is stationary. This seemed to be confirmed by the observation that if we jump in the air, the earth does not spin away beneath us. Today, we know that the earth moves, spinning on its axis and orbiting the sun and we accept that the earth moves at more than 100 meters per second and that inertia explains why, after jumping, we land where we took off. What has happened is that nowadays we interpret our observation of being apparently stationary in a different way because of a new conceptual framework derived from our improved scientific knowledge. In the same way, we have learned to doubt the validity of certain observations such as the well-known moon illusion that makes the moon appear much smaller in the sky than when it is low on the horizon [160].

Instead of relying on simple observations for determining what the “facts” are, scientists accept that reliable facts can usually be obtained by performing experiments that are always theory-dependent and require that we make certain inductive inferences. When we extrapolate from a finite number of experimental observations and make a general scientific claim, we accept that this induction procedure only allows us to reach approximate or probable scientific truths but never the type of “proven” truths achievable by logical deduction.

Scientific observations do not only refer to objects, properties and events that we can observe using our senses but also concern the observation of theoretical entities that are less accessible to direct human experience. Certain objects or phenomena may simply be non-observable because they lie outside the range of objects that our senses can directly observe, and therefore require instruments such as microscopes or telescopes. Scientists sometimes also need to observe theoretical entities for instance epitopes or paratopes that can only be defined structurally or functionally on the basis of certain criteria such as a minimum contact distance separating two interacting partners, or the occurrence of a binding reaction between them. In such cases, making a distinction between observable terms and theoretical terms may become difficult and this has led to the claim that all scientific observations may be theory-laden [163] (pp. 41–61).

Although experimental observations may not on their own justify a belief in the reality of invisible entities such as epitopes, it is our ability to successfully manipulate them empirically that convinces us that they actually exist. As pointed out by Hacking [155], we need to interfere with the material world in order to obtain knowledge about it and our scientific understanding will increase when we are able to intervene in a system and can manipulate it successfully.

A scientific theory refers to a well-substantiated body of explanations, repeatedly tested and confirmed experimentally, which have so far overcome all attempts to falsify them and allow accurate predictions to be made. Theories, therefore, should not be easily dismissed and paradigms are likely to be useful only if they are not contradicted by accepted scientific theories. In everyday conversation, the term theory is often used pejoratively as being a wild guess not to be taken seriously (i.e., this is just a theory), but this is not the case for well-established scientific theories that have become synonymous with well-established scientific facts. For instance, Darwinian evolution is today considered a scientific fact and no longer just a theory, as argued by the adepts of intelligent design.

Scientific explanations usually contain laws in the form of causal explanations that link causes and effects. Causation is a much stronger relation between events than mere temporal succession since the effect is unavoidable and follows the cause necessarily and not only regularly. However, what constitutes the actual link in a causal sequence remains highly mysterious and is an unsolved problem in the philosophy of science. Gravity, for instance, remains mysterious because it does not need any material connection in order to be transmitted. 

Causation in the physical and biological sciences possesses very different explanatory power. In physics, only a small number of physical forces exist that cause observable phenomena, and the occurrence of an eclipse, for instance, can be explained using Newton’s laws of universal gravitation together with certain initial conditions. These laws on their own suffice to describe the motions of celestial bodies in an interstellar vacuum where there are no external interferences. On Earth, these laws explain physical phenomena only if we add a *ceteris paribus* clause (i.e., other things being equal) to rule out interfering factors such as electrostatic or magnetic forces [164]. The very accurate predictions achievable in astronomy are not possible in the case of complex biological systems that always present an enormous diversity of interactions between their numerous constituents and give rise to network behaviour absent in the isolated components [165]. Complex systems also possess emergent properties that have their own causal powers not found in the individual constituents of the system and these properties cannot be predicted by a reductionist analysis of the individual components of the system [156,159,166]. However, it is nowadays accepted that we need not achieve a full theoretical understanding of a complex biological system in order to be able to make some reliable predictions about how it is likely to behave. This means that we might be able to achieve protective immunity by vaccination without necessarily understanding all the multiple underlying mechanisms at work in a human immune system.

### 7.2. Degeneracy of the Immune System and Polyspecificity of Antibodies

The term degeneracy was introduced in the life sciences to refer to the fact that the genetic code is “degenerate” or “redundant” because there are many more triplet codons than encoded amino acid residues [167]. As a result, a huge number of distinct mRNA sequences can be translated to generate the same protein sequence. Degeneracy is a common feature of complex biological networks, where structurally different elements are able to perform the same activity or function. In immunology, degeneracy refers to both the ability of structurally different Abs, BCRs, or T cell receptors (TCRs) to bind to the same ligand and to the ability of a single Ab or T cell receptor to bind many different antigens or peptides [124,168]. 

Degeneracy is a property of the IS as a whole and not of its individual components [125] whereas the ability of an individual Ab to bind different antigens is described as cross-reactivity, molecular mimicry or promiscuity. This means that there is no unique intrinsic epitope for any Ab molecule but only a diverse group of potential ligands [126]. The degeneracy of the IS makes it possible for a limited number of immune receptors to recognize, albeit often with very low affinity, essentially any molecular motif that an animal’s IS is likely to encounter. Specificity has been defined as the exact complementary relationship between an agent and something acted on [169] a definition that applies to the stereochemical complementarity between antigen and Ab, enzyme and substrate or receptor and ligand. Specificity in immunology has been difficult to define [170,171] and in the case of Abs, it has been suggested that it may be better to refer to their discriminating capacity rather than to their specificity since it is only the wish of an investigator to distinguish between two antigens that determines whether an Ab is considered to be specific or not [172] (p. 291) [173,174] (p. 42).

Whereas Ab affinity describes the strength of the binary relationship between one epitope and one paratope, Ab specificity is a ternary relational property that has meaning only with a minimum of three partners, for instance one paratope and two epitopes. There is no necessary link between affinity and specificity because antibodies of low affinity are often able to better discriminate between two antigens than antibodies of high affinity that tend to reveal more cross-reactions [173]. In order to evaluate the efficacy of a vaccine, it may be necessary to measure the affinity of the Abs that it elicits and this can be done, for instance, by calculating Ab avidity indices obtained by disrupting Ab-HIV-1 Env complexes with chaotropes [175]. Ab binding to continuous epitopes resists chaotrope-induced disruption more than Ab binding to the more fragile discontinuous epitopes which, however, are the epitopes that induce most bnAbs [176]. There is only limited correlation between Ab affinity and chaotrope resistance because of the phenomenon of hysteresis, which occurs because the energy of Ab-antigen dissociation is higher than the energy of association [177] (pp. 99–125).

It is therefore preferable to assess Ab affinity by measuring the kinetics of Ab-HIV-1 Env interactions using surface plasmon resonance biosensors, a technique that also allows the determination of the active concentration of the reactants [178] and provides kinetic constants that are better correlated with Ab neutralizing capacity than the avidity indices obtained by chaotrope disruption [179,180] (pp. 805–828).

**Figure 4:**
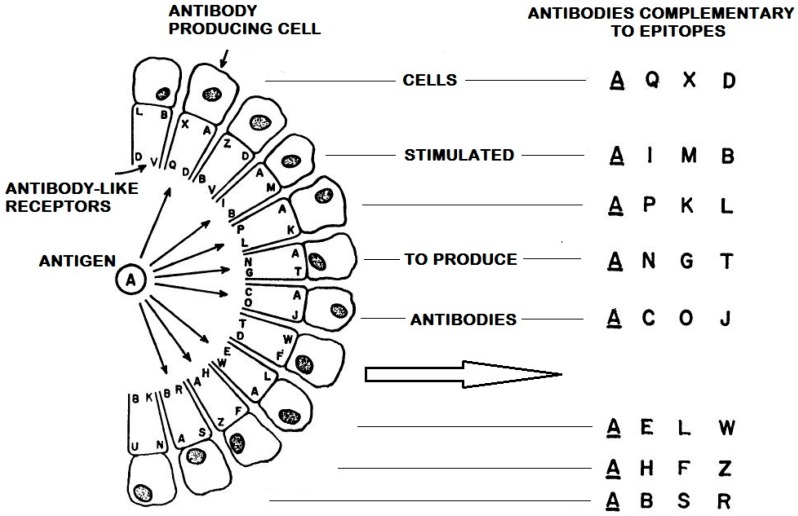
Immune serum specificity as a population phenomenon. Individual B cell receptors are shown as having properties similar to immunoglobulin combining regions. For illustrative purposes, these are drawn as being each complementary to four different epitopes or antigens; we suppose that this number is in fact much larger. Stimulation by antigen A causes the cells with A specificity to divide and produce antibodies directed against A. The immune serum produced will therefore react in high titer with antigen A. Each produced immunoglobulin also has other specificities, but because these need not be the same in every molecule, the other specificities, B to Z, will be diluted out and will react only in low titer (from Richards et al., 1975 [182]).

It may seem counterintuitive that the polyspecificity of Abs is in fact responsible for the greater collective specificity of a polyclonal antiserum induced by a multi-epitopic protein compared to the discriminating capacity of a single Mab [181,182]. Since all the Abs in a polyclonal antiserum are polyspecific, they will all react with many unrelated epitopes, but as these cross-reactive epitopes are all different for each Ab in the antiserum, the cross-reactive potential present in the serum will be diluted out and will be masked (Figure 4). Such a dilution effect does not occur in the case of a Mab and its ability to cross-react with other epitopes is therefore more easily observed [29]. The same phenomenon explains the remarkable specificity of the multi-epitope ligand cartography technique that uses Mabs conjugated to different fluorescent dyes for detecting the topological location of individual proteins in multimolecular assemblies present within cells and tissues [183,184]. 

In structure-based RV, the Mab used to characterize one HIV-1 epitope by crystallographic analysis of a Mab-Env complex is always polyspecific, which means that this epitope is only one of the several epitopes that the Mab is able to recognize. When that Mab is present as a BCR in the immunized host, it could also have been selected by other cross-reactive epitopes rather than by the one elucidated by crystallography. This means that this structurally defined epitope does not necessarily correspond to the immunogenic structure believed to be active during the immunization process. Since an Ab is never monospecific for a single epitope, there is no reason to assume that a particular Ab can only be elicited by a single immunogenic, epitope structure. This is one of the reasons why a reductionist structure-based RV approach that concentrates on only one epitope is unlikely to be able to reveal the structure of effective vaccine immunogens capable of inducing polyclonal immune responses [130,142].

Since Abs are also heterospecific, i.e., often able to react much better with other antigens than the one that was used in the immunization process used to elicit them, the immunogenicity of an epitope is also not necessarily accompanied by an antigenic reactivity that will enable it to bind to the induced Abs [29]. Antigenicity and immunogenicity are thus not properties that are always present simultaneously in the same regions of a protein molecule. This underlines the need to distinguish the binding capacity of an antigen or epitope, i.e., its antigenicity, which is a chemical property, from its immunogenicity, which is a biological property determined mainly by mechanisms and factors in the IS that are independent of the chemical basis of epitope–paratope recognition—the only parameter considered by reverse vaccinologists.

### 7.3. Structural and Functional Analyses Delineate Different Epitopes

It has been known for a long time that structural and functional approaches to the study of protein antigenicity lead to different perceptions of the nature of protein epitopes [79,185] (pp. 55–79).

Functional studies take the form of Ab-antigen binding measurements that tend not to provide reliable information on which residues in the antigen and Ab molecules make contact with each other since the conformation of the interacting molecules depends considerably on the format and conditions of the binding assay that is used. When the results of liquid-phase and solid-phase immunoassays are compared it may lead to the conclusion that different residues in the two partners participate in the two types of assay [186]; a similar uncertainly prevails when the antigenic peptides are tested in their free form or conjugated to a carrier [187]. When longer antigenic peptides are found to bind better in an assay that shorter ones, it is difficult to know if the additional residues present in the longer peptide actually bind to the Ab or simply induce a conformation that is better recognized by the Ab. It is also sometimes observed that when the length of an active peptide is increased, this completely abolishes its ability to bind to an Ab [187]. It is difficult to interpret in structural terms the results of functional binding assays with both continuous and discontinuous epitopes since such assays are rarely able to clearly establish which amino acids are directly involved in an interaction. These assays therefore are more useful for providing information on the affinity and biological activity of the reactants than on their structure [188]. 

It is undeniable that studies of the structure of protein epitopes involving the crystallographic analysis of Ab-antigen complexes provide a clear picture of what epitopes consist of. Initially structural studies were interpreted using a lock-and-key model which assumed that the binding process did not lead to significant conformational changes in the two partners [189]. This model was later abandoned when structural studies of antigen and Ab molecules in their free and bound state demonstrated that binding usually involves a process of induced mutual complementarity and fit resulting from important side-chain movements and changes in the backbone conformation of the two partners [44,45,190]. Since the structures visualized in Ab–antigen complexes usually differ considerably from the structures of the unbound binding sites, it is in fact impossible to know exactly which epitope structure was initially recognized by the Ab molecule when it was in its free form or present as a BCR at the surface of a lymphocyte in the host IS. Although somewhat different criteria can be used for inferring which residues are in contact with each other at an antigen-Ab interface [39,191] crystallographic elucidation of the complexes tends to promote a deceptive perception that epitopes and paratopes are fairly static patches of interacting surface residues. Space-filling models of protein surfaces show many juxtaposed neighboring atoms and this may obscure underlying secondary structure elements and give the impression that two static interacting units are in contact. Such an interpretation of epitopes and paratopes as static interacting surfaces is, however, refuted by the fact that paratopes are constituted of flexible CDR loops that allow them to adapt to a variety of epitopes, which themselves possess considerable flexibility [123,192,193,194] and are often located in regions of high segmental mobility in the protein sequence [12,136,195,196,197].

The HIV-1 V3 antigenic site is a particularly good example of the difficulties encountered when a well-delineated structural element of the Env spike is analyzed for its ability to react with neutralizing Abs found in HIV-1 infected individuals or in immunized humans and animals. The semi-conserved and flexible loop V3 plays a major role in the recognition of chemokine co-receptors by the virus and deleting the V3 region is known to completely abrogate virus infectivity [198]. The V3 loop has a constant size of about 35 residues and has for many years been considered as the principal neutralizing domain of the virus [199,200]. Anti-V3 antibodies are able to block virus infectivity by interfering with co-receptor recognition [201,202] although the value of V3 as a potential vaccine candidate has sometimes been questioned because no anti V3 antibodies appeared to be present in anti-HIV-1 antisera when these were tested with linear V3 peptides [203]. It was subsequently demonstrated that these antisera did actually contain anti-V3 antibodies that only reacted with constrained cyclized V3 loops possessing the correct conformation not present in linear V3 peptides [202,204,205].

**Figure 5:**
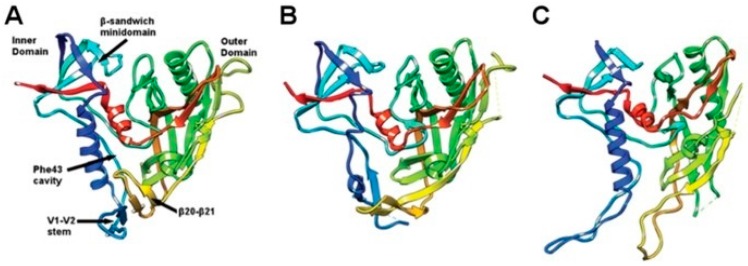
Structure of gp120 in different ligation states. (**A**–**C**) Comparisons of gp120 structures in (**A**) the CD4-bound state; (**B**) the b12 antibody-bound state; and (**C**) the F105 antibody-bound state. Structures are rendered as ribbon diagrams colored spectrally along the sequence from blue at the N-termini to red at the C-termini. The view is into the face occupied by CD4 interactions. (Figure from Korkut & Hendrickson [211].)

The V3 loop is very flexible and can adopt different conformations when it binds to different Mabs [206], making it difficult to ascertain which conformation is likely to be optimal for a V3 synthetic peptide vaccine. NMR studies of V3 loops bound to Abs specific for different HIV-1 isolates, revealed that the conformation of Ab-bound V3 peptides was dictated by a process of induced fit to each Mab [207] and thus that the observed structure of a bound V3 epitope did not indicate which immunogen conformation was likely to elicit a particular type of neutralizing Ab (Figure 5). Even cyclic V3 peptides retain significant flexibility and although V3 loops can be chemically constrained in particular conformations [208,209] there is no evidence whether better vaccine candidates would necessarily be obtained by increasing the rigidity of the V3 loop that is naturally flexible and is therefore more easily recognized by a variety of BCRs [210].

Recent crystallographic analysis of the trimeric pre-fusion HIV-1 Env showed that V3 loop residues are occluded in a pocket, formed by the V1V2 loop and V1V2 stem of its own protomer and by the V1V2 stem of the neighbouring protomer, giving rise to quaternary interprotomer interactions [212]. As a result the V3 loop is poorly accessible to anti V3 antibodies [213]. A recent study [214] showed that individual mutations in various regions of the Env-trimer induce an open conformation that exposes the V3 loop to V3 Abs and lead to virus neutralization. It had previously been shown that if V3 Mabs were incubated with the virus for a long time before adding target cells in a neutralization assay, the neutralization capacity of V3 antibodies was considerably enhanced, suggesting that dynamic changes in the V3 loop accessibility were required for optimal neutralization [215]. There is considerable evidence that the trimeric Env spike possesses considerably more flexibility than previously thought and that this plasticity is also present in the unliganded state of the trimer. Env promotes entry of the virus into target cells by recognizing the cellular CD4 receptor and this leads to conformational rearrangements in Env, which allow binding to the co-receptor binding site and triggers the fusion of viral and cellular membranes and mediates virus entry in the host cell [21,216].

By incorporating fluorophores into unliganded HIV-1 Env trimers on the surface of native virions, it was possible, using fluorescence resonance energy transfer (FRET) methods, to demonstrate that the trimers fluctuated between three distinct conformations [217,218]. A low-FRET state reflected the closed ground state conformation of the prefusion Env while two higher-FRET states could be stabilized by soluble CD4 and by the co-receptor-mimicking antibody 17b. Since all three FRET states were also observed in the absence of ligands, the conformations that are stabilized by CD4 and co-receptor could be studied with unliganded HIV-Env, demonstrating an unexpected, considerable intrinsic structural flexibility in the Env protein. These new findings helped to clarify some of the unexplained features of the neutralization behaviour observed with certain nMabs that could be the result of large scale conformational transitions in gp120 [211,214]. 

Crystal structures of gp120 bound to Fab fragments of Mabs b12 and F105 and to CD4 have demonstrated the extensive rearrangements of gp120 in different ligation states (Figure 5) and shown that intermediates generated from antibody-bound states deviate from the initial structure more than those generated from the CD4-bound state, highlighting the crucial role that conformational masking of epitopes plays in allowing the virus to escape the IS [211]. The extreme plasticity of the HIV-1 Env will no doubt continue to pose considerable challenges for developing a vaccine aimed at a virus that exhibits such enormous antigenic variability and structural plasticity.

### 7.4. The Immunogenicity of a Protein Molecule Is a Complex Biological Property that Cannot Be Predicted from the Molecule's Antigenicity, Which Is Simply Its Ability to Bind Certain Immunoglobulins

An epitope can be defined as any accessible part of a protein that can be recognized by a paratope present in a free Ab molecule or in a BCR at the surface of certain B cells. It must be emphasized that this epitope definition does not include an immunogenic capacity to induce, in different immune systems, the same Ab that the epitope is able to react with; if this were the case, the existence of an epitope in a protein would depend on immunogenetic and immunoregulatory mechanisms of the immunized host. If a molecular biologist engineers a new protein that previously did not exist and therefore was never used to elicit Abs, it would nevertheless possess epitopes able to cross-react with Abs previously induced by other proteins.

Jay Berzofsky in a much cited review published more than 30 years ago [136] clearly distinguished between properties intrinsic to protein antigens (such as their chemical and physical structure) and other properties of the host IS that are extrinsic to the antigen molecule but control its immunogenicity, such as the host antibody gene repertoire, the specificity of helper and suppressor T cells, antigen processing, self-tolerance, idiotypic networks, and many additional immunoregulatory mechanisms. Of all the potential epitopes of a protein, only a subset will therefore be immunogenic in any individual host.

The difference between antigens and immunogens is crucial in vaccinology but is often not sufficiently appreciated. Most peptides are immunogenic since they readily elicit Abs that react with the peptide immunogen [219] (pp. 113–177). In the context of vaccination, however, what is relevant is so-called cross-reactive immunogenicity which is the ability of epitopes to induce Abs that cross-react with the parent native protein, as well as cross-protective immunogenicity which is the ability of epitopes to induce Abs that neutralize the infectivity of the pathogen harboring the antigen [68]. It is of course the IS that elicits and produces antibodies and not the antigen or immunogen which only plays a triggering role. It is important to appreciate that the chemical environment is not the same when an epitope recognizes either a free Ab molecule or a BCR embedded in a lipid membrane. This means that the antigenic epitope bound to a free Ab may not be identical with the immunogenic epitope that interacts with a BCR [18,220]. When an HIV-1 peptide reacts in an immunoassay with an Ab directed to a native protein, the Ab may select one of the many conformations present in the peptide or it may induce a reactive peptide conformation by induced fit. On the other hand, when the same peptide is used as an immunogen, different peptide conformations may be recognized by separate BCRs and there is no reason why the peptide should bind preferentially to those rare receptors that either cross-react with an epitope present in the native protein or that will subsequently lead to the secretion of neutralizing Abs by plasmocytes [46,131,221].

The selection of BCRs during immunization is blind to the presence of neutralizing anti-viral activity in the Abs that will subsequently be secreted since the selective forces operating during immunization depend mainly on reactant concentration and on the probability of lymphocyte stimulation occurring above a certain triggering threshold [222,223].

## 8. Conclusions

Many hypotheses and theories have been proposed to explain the evolutionary origin of the IS. Initially, the IS was considered to be a defense system against pathogens and invaders and subsequently this gave rise to the concept of the Protecton [224]. Later on, it became generally accepted that the main purpose of the IS was to discriminate between self and non-self, although there is no generally accepted definition of immune selfhood [225]. In 1974 Niels Jerne proposed his idiotypic network theory [226] according to which the IS is a network of anti-idiotypic Abs that recognize epitopes called idiotopes present on the variable regions of other Abs in the same individual. Such Abs are produced in an IS because the initial Abs1 induced by an external antigen act as functional self-antigens that induce secondary Abs2 directed against idiotopes present in the Abs1. An Ab2 could mimic the epitope recognized by Ab1 and may correspond to an internal image of the external antigen. Subsequently, tertiary Abs3 directed against the Abs2 may appear leading to a cascade of anti-idiotypes which transforms the IS into a self-regulatory network of anti-idiotypes [185] (pp. 55–79). Such a cognitive system acts as a regulatory network of anti-idiotypic Abs, which stabilizes the antigenic integrity of the body and prevents the immune response from getting out of control or producing autoimmune disease [125,222,225]. Once it was recognized that all Abs, BCRs and TCRs are polyspecific and that autoimmunity is a natural, normal immune phenomenon [106,225], other theories favoring immune incorporation instead of interception were developed [125] which gave rise to intense debates between the proponents of various interpretations of immune activity [227].

In the title of the present review, reference was made to the fact that an approach or procedure used to develop a vaccine should not be incompatible with accepted immunological theories. However, none of the theories about the origin and nature of the IS mentioned above are universally accepted and immunologists disagree about which theory will eventually gain general acceptance. On the other hand (see Section 7.1), other immunological theories have become widely accepted and since they have overcome all attempts to falsify them, such theories should not be ignored by vaccine developers. For instance, the degeneracy of the IS and the polyspecificity of Abs and BCRs are scientific facts that must accepted by vaccine developers and, since they are not compatible with the presuppositions underlying structure-based RV, it is not astonishing that this approach failed to yield an effective HIV-1 vaccine. The failure of structure-based RV can be explained as follows [131,142]:

1. The epitope structure observed in a complex when it is bound to a neutralizing, polyspecific Mab is only one of the many epitopes that can be accommodated by that Ab and there is no reason to assume that it must correspond to the immunogenic epitope that elicited the Mab.
2. The structural delineation of HIV-1 epitopes bound to neutralizing Mabs cannot tell us which vaccine immunogens will be able to induce a protective polyclonal immune response. Improving the antigenic binding capacity of an epitope does not constitute vaccine design.
3. Short continuous epitopes of HIV-1 that are mostly only a part of more complex discontinuous epitopes of the virus are not a suitable starting material for developing effective vaccine immunogens.
4. Rational design can be used for optimizing the antigenic binding capacity of one HIV-1 epitope for one nMab or for improving the reactivity of one nMab intended for passive immunotherapy. However, structure-based rational design for improving the binding capacity of one epitope or one paratope does not constitute vaccine design and it cannot elevate vaccine research from an empirical exercise to a scientific discipline. Empirical experimentation aimed at manipulating the immune system successfully is entirely rational and is the approach that was successfully used in the past for developing effective vaccines.
5. Since a complex biological system like the IS possesses many emergent properties that are not present in the individual components of the system, it is not possible to fully understand and control the IS using the reductionist strategy of dissecting and analyzing separately the structure, activity, and interactions of all its numerous constituents.

The RV paradigm has guided a considerable part of HIV-1 vaccine research for about a decade although it completely failed to produce an effective vaccine immunogen. Hundreds of RV research projects aimed at increasing our basic knowledge of HIV-1 immune responses and immunopathology were funded which produced a remarkable harvest of basic science publications that described in detail the structure of many new HIV Env epitopes and their complementary Ab paratopes. This large body of new factual knowledge may one day be useful for passive immunotherapy purposes in spite of the fact that it did not help the development of a vaccine intended for active immunization. This situation once more illustrates the principle that the prescriptive knowledge that vaccinologists require in order to successfully intervene in an immune system and achieve protective immunity is not simply derived from previously acquired factual knowledge obtained by basic research. As explained in Section 6, a new HIV-1 vaccine is always an invention that necessarily involves an empirical, trial-and-error step that inherently differs from a rational design approach; in the case of HIV vaccines the so-called rational design approach was also ineffectual because it confounded immunogenicity with antigenicity.

Referring to HIV-1 epitopes as vaccine immunogens able to elicit bnAbs probably also added to the confusion since it encouraged investigators not to focus on the intrinsic complex features of the host immune system which hold the key to the development on any vaccine. The past unproductive RV episode that did not help the development of an HIV-1 vaccine could possibly be valuable if it reminds future investigators that they should not disregard established scientific theories in their search for the prescriptive knowledge needed for developing any vaccine.
